# The “Real Welfare” Scheme: Changes in UK Finishing Pig Welfare since the Introduction of Formal Welfare Outcome Assessment

**DOI:** 10.3390/ani12050607

**Published:** 2022-02-28

**Authors:** Fanny Pandolfi, Claire Barber, Sandra Edwards

**Affiliations:** 1 Independent Researcher, 75000 Paris, France; 2 Animal Health & Welfare, Agricultural and Horticultural Development Board, Kenilworth CV8 2TL, UK; claire.barber@ahdb.org.uk; 3 School of Natural and Environmental Sciences, Newcastle University, Newcastle upon Tyne NE1 7RU, UK; sandra.edwards@newcastle.ac.uk

**Keywords:** enrichment, hospitalization requirement, lameness, lesions, pigs, tail biting

## Abstract

**Simple Summary:**

Farm animal welfare is an important issue for both farmers and the general public. In order to monitor animal welfare on a large population of finishing pig farms in the UK, the “Real Welfare” project developed a methodology for on-farm assessment based on regular measurement of some key animal-based measures, so-called welfare outcomes. This paper presents estimates of the percentage of these different welfare outcomes over the years since the inception of the scheme. Between 2016 and 2019, the mean percentage of pigs in the mainstream herd showing four mandatory welfare outcomes (pigs requiring hospitalization, lame pigs, pigs with severe tail lesions, and pigs with severe body marks) was very low and a decreasing trend was observed for all outcomes except severe tail lesions. These data give good representation of the overall situation in the UK finishing pig population.

**Abstract:**

Farm animal welfare is an increasingly important issue, leading to the need for an efficient methodology to deliver accurate benchmarking. The “Real Welfare” project developed a methodology based on regular recording of a limited number of animal-based measures, so-called welfare outcomes, which allows faster and easier on-farm assessment of finishing pig welfare. The objective of this paper is to estimate, with sufficient robustness and confidence, the prevalence of different mandatory and optional welfare outcomes in the mainstream herd of the finishing farms in the UK based on the “Real Welfare” scheme data and to assess the changes in prevalence over time, inspection visits and seasons. The mean overall prevalence of the four mandatory welfare outcomes (pigs requiring hospitalization, lame pigs, pigs with severe body marks, and pigs with severe tail lesions) was very low (≤0.2%) and a significant decreasing trend was observed for the first three of these mandatory welfare outcomes since the inception of the scheme. This result might reflect either a reduction in factors giving rise to welfare problems in the mainstream herd or increasing awareness about management of compromised pigs. Additional data are required to clarify these possibilities, but both represent improved pig welfare.

## 1. Introduction

As a result of a growing body of scientific research and awareness-raising campaigns, farm animal welfare has become an increasingly important issue for consumers, leading to progressive developments in product marketing and in national and European legislation [1,2]. These changes have highlighted the need for an efficient methodology to accurately benchmark animal welfare. There has been a growing trend in the adoption for this purpose of animal-based measures, sometimes called welfare outcomes (WO), which are now recognized as a better alternative to assess animal welfare compared to the assessment of features in the environment [3,4]. The EU Welfare Quality^®^ project pioneered the development of a methodology for on-farm assessment of animal welfare with animal-based measures [5]. However, the large set of measures and the time required for completion of their comprehensive protocol made it impractical to utilize for a routine assessment which could be applicable for repeated regular use on a large sample of farms. The creation of simpler alternatives became possible thanks to the development of a limited number of “iceberg indicators”, which can be defined as single measures which are reflective of several different aspects of animal welfare. These act as a signal of multiple health or welfare issues and, therefore, allow faster and easier on-farm assessment [6].

Previous studies have indicated that the prevalence of such WO is often low [3,7,8]. Consequently, accurate estimation of the prevalence of different WO requires a very large sample which is also representative of the population of interest [2]. Such large samples are particularly difficult to obtain in scientific research based on primary data analysis, due to the difficulty to enroll a large number of farms, the restricted time available and evident cost limitations. Secondary analysis of the data arising from mandatory inspections or schemes which encompass a large part the national herd (i.e., analysis of large datasets not initially collected for the purpose of a study) might therefore represent a good alternative to accurately estimate the current status and trends in health or welfare indicators with low prevalence.

The “Real Welfare” scheme was developed in order to assess pig welfare on finishing farms using animal-based measures [9]. These “iceberg indicators” were chosen after stakeholder consultation to capture the most important welfare issues for the pigs and the industry, using protocols developed and piloted in a previous research project [9]. The “Real Welfare” scheme data represent a unique database which is representative of the mainstream herd of finishing pig farms in the UK and now includes data collected on repeat visits over many years. The results presented in this paper represent the third in a series of publications based on a secondary analysis of the “Real Welfare” scheme data. The first paper focused on the description of the methodology for collection of “Real Welfare” data and the assessment of the initial trends in the prevalence of the welfare outcomes during the period of introduction of the scheme [9]. The second paper described farm characteristics and the associations between farm features and management and the prevalence of the different welfare outcomes over this initial period [10]. The objective of this third paper is to extend the analysis to the period after full stabilization of the scheme, estimating the long-term trends in the prevalence of different WO and the extent to which they can be considered as representative of the mainstream herd of finishing pigs in the UK. It includes assessment of the trend over time and the changes over successive farm inspection visits and seasons of the different WO for the whole six-year period (2013 to 2019) covered by the scheme. To further extend the information presented in the previous papers, we combined different methodologies to increase the robustness of our analysis and conducted a power analysis to estimate the confidence in the trend of the prevalence of the different WO and to assess to possibility to extend the results to the mainstream herd of all finishing farms in the UK.

## 2. Materials and Methods

Based on the data described below, we made basic descriptive analyses of the number of farms, pens and pigs assessed in this study and described the prevalence of different WO which have been measured during the assessment. Moreover, we used specific statistical analyses to measure the association between different WO and the changes of these WO over time. Additional precision analysis was carried out to strengthen our analysis and its interpretation.

### 2.1. Data

**Figure 1:**
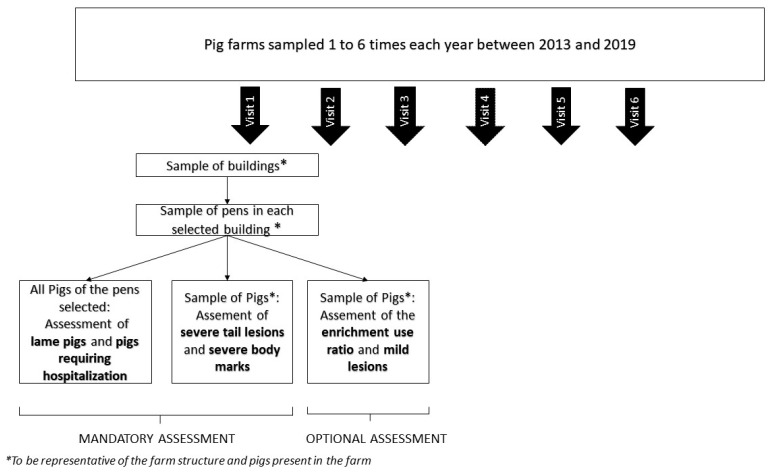
Flowchart of the yearly “Real Welfare” scheme assessment between 2013 and 2019.

The analyses in this report were carried out using all data collected from on-farm assessments conducted in the context of the “Real Welfare” scheme between the 4 April 2013 and the 31 December 2019. This includes the data collected between 2013 and 2016 used for the two previous publications [9,10] and additional data collected from 2017 to 2019. Some farms were followed over the whole period covered by the scheme, whilst others were present only during a part of this period. The farms assessed by the “Real Welfare” scheme produce approximately 95% of all commercially raised pigs in the UK [11]. A random sampling of pens and pigs was conducted during each visit by a trained vet, targeting to be representative of the pig herd (Figure 1, Supplementary Material S1). An earlier publication, based only on the farm visited between April 2013 and May 2016, described in detail the training of veterinarians performing the assessment, the sampling methodology, the standardized on-farm assessment protocol, and data collection, all of which are owned and managed by the Agriculture and Horticulture Development Board [9]. Earlier publications also described the farming systems (indoor pens exclusively, both indoor and outdoor pens, outdoor exclusively), group size (small (<30pigs), medium (30–200 pigs), large (>200)) and the percentage of tail-docked pigs for the farms of the “Real Welfare” scheme [9,10]. Two databases were used to conduct our analysis. These comprised a database at the farm level, with information from 2616 different farms, and a database at the pen level, with information from 253,713 different pen inspections. In total, 182 pens were excluded from the analysis because they lacked a unit code (farm identification). Data were collected only from production pens, with hospital pens excluded, as was the case for the Welfare Quality^®^ scheme from which the protocol of the “Real Welfare” scheme was derived [5]. This was because the focus was placed on the identification and appropriate treatment of welfare problems in mainstream pens (excluding hospital pens). From the date of the assessment, the calendar year and the season were extracted. Four seasons representing spring (March, April, May), summer (June, July, August), autumn (September, October, November) and winter (December, January, February) were identified from the date of assessment.

In total, nine different WO were recorded for some period of time at the pen and farm levels: pigs requiring hospitalization (pigs who are sick, injured or lame and are unable to compete for resources, being bullied/tail bitten or would benefit from access to bedding that is more comfortable than that available in the mainstream pen), lame pigs, pigs with severe tail lesions, pigs with severe body marks, enrichment use ratio (number of pigs interacting with enrichment/number of pigs interacting with enrichment or other pen features or pen mates), pigs with mild tail lesions, pigs with dirty tail, pigs with mild body marks, and pigs with dirty body. The enrichment could be natural enrichment (straw, wood) or objects [9,10]. The recording of tail lesions and body marks changed during the implementation of the “Real Welfare” scheme. After an initial 8 months period, a review of the scheme’s operation determined that recording the enrichment use ratio, minor tail lesions (dirty tails and mild tail lesions), and minor body marks (dirty bodies and mild body marks) should become optional to facilitate the implementation of the scheme and focus on the most important WO to assess pig welfare. Minor lesions and enrichment use were consequently recorded for only a subsample of farms between January 2014 and December 2019. In total, 32,024,840 pigs were present on the farms visited between 2013 and 2019. In order to assess the percentage of pigs requiring hospitalization and lameness, 13,678,660 pigs were assessed individually, while 7,112,777 pigs were assessed individually for tail lesions, and 7,110,608 for body marks. In total, 65,023 different pens grouped in 10,549 different buildings were assessed. However, as some pens were visited several times and were considered different between the different visits, the actual number of pen visits between 2013 and 2019 was 253,713.

For each type of lesion, the total number of pigs assessed consisted of the number of pigs with a specific lesion and the number of pigs in the same pen without this lesion. This allowed calculation of the percentage of assessed pigs exhibiting a given WO in each pen and each farm. The enrichment use ratio records the proportion of actively investigating pigs in the pen which are directing this behavior to the enrichment provided rather than to other pigs or pen components and was expressed on a 0–100 scale.

### 2.2. Number of Pigs, Pens and Farms Visited

An initial description was made of the sample available for the analysis: number of pigs, farms, buildings and pens, number of assessment visits, and average herd size for the full period of the “Real Welfare” scheme. We also described the average percentage of pigs assessed per visit (based on the number of pig places) and the average number of pens and buildings assessed per visit. For 10 farms, the number of pigs sampled exceeded the number of pigs reported as present in the farm, then the percentage of pigs assessed was replaced by a missing value.

Given that farms and pens were visited several times, the total number of pens assessed over the entire period may have included the same pen numbers and farm IDs multiple times, but they were treated as different pens for the descriptive analysis because the pig population would be different at each visit. Moreover, the farms were generally visited several times, but the same pens were not always sampled.

### 2.3. Associations between the Different Welfare Outcomes and Prevalence

The sample size of “Real Welfare” scheme data, the population coverage and the random sampling applied at the pen and pig levels allowed calculating estimates for the mean percentage of the different WO of the mainstream herd of finishing pig farms in the UK with 95% confidence interval. The mean percentage was estimated based on either the proportion of pigs with lesions among all pigs in the pen (for lame pigs and pigs requiring hospitalization at the pen level) or the proportion of pigs with lesions among a sample of pigs present in the pen or the farm (Supplementary Material S1). The mean percentage, standard deviation, median, minimum and maximum values, 10% and 90% percentiles at the pen level, and the mean, minimum and maximum values for the annual rolling average per pen are presented for each WO. The mean percentage, standard deviation, median, minimum and maximum values, 10% and 90% percentiles were also calculated at the farm level based on the annual rolling average of each farm. The number of pens assessed per year for mandatory and optional WO were separately calculated.

In order to understand the associations between the different WO at the pen level, the correlations between them were calculated using Spearman’s rank correlation. The correlation was considered significant if *r* > 0.3 and *p* < 0.05 [12]. For mandatory WO, the correlations were calculated for the whole sample. The correlation between all WO were calculated only for pens for which optional WO were recorded.

### 2.4. Assessment of the Changes over Time, Visits and Seasons of the Different Welfare Outcomes

Two complementary approaches were used to assess changes in the prevalence of the different WO over time: time series analysis, and regression. Time series analyses were conducted for the WO in order to identify an underlying trend in the data. We converted the data at the pen level in monthly time series for all WO. The time series was decomposed into three components, which were the underlying trend component, a seasonal component (pattern that repeats with fixed period of time) and an irregular component (residuals after allocation into trend and seasonal components). Visual interpretation of the plot was used as well as the augmented Dickey–Fuller (ADF) test (unit root test) and autocorrelation function (ACF) to assess, respectively, the stationarity and the autocorrelation of the time series (Supplementary Methods S1).

Additional analyses were conducted to identify associations between specific years, calendar seasons or visits and the prevalence of WO, taking into consideration the data structure associated with the specific farm and assessor identity. The changes over calendar years in the mean values of all the measures of welfare were assessed with generalized linear mixed models (glmm) (one for each WO) in an analysis performed at the pen level. The dependent variable was the proportion of pigs exhibiting each WO, or the enrichment use ratio. The variable ‘year’ was considered as a fixed effect. The pen nested in the farm unit was considered as a random effect as different pens could belong to the same farm. The interaction between the farm unit and the vet practice that performed the assessment was also used as a random effect in order to consider any potential inter-observer variations. In order to make all the pair-wise comparisons between years, we used Tukey’s Honest Significant Difference (Tukey’s HSD) method and the Compact Letter Display (CLD) function was applied to the results from Tukey’s HSD method to identify sets of years which were detectably different (Supplementary Methods S1). The same analyses were performed with ‘calendar season’ as the fixed effect instead of year for the mandatory WO and the enrichment use ratio.

Since farms entered and left the “Real Welfare” scheme at different points in time, the changes over successive inspection visits for each individual farm were also assessed. The mean percentage of pigs exhibiting each WO was calculated for each farm visit (from the first to the last date of the visits). Based on this value, the changes over the visits were assessed using Kendall’s tau b correlation between visits for each individual farm and the mean percentage for all farms for each visit was plotted on a graph in order to visualize the general trend of the changes that occurred with the progressive number of assessment visits.

### 2.5. Precision of the Estimates and Trend over Time

Firstly, to calculate the precision of the estimates and trends over time, the sample size was adjusted for each year in order to consider the design effect.

- The intraclass correlation (ICC) for each welfare outcome was calculated with the R package ICC.
- The design effect (Deff) was calculated based on the ICC and the average number of pens per farm (m) as follows:

Deff=1+ICC(m−1)

- The estimated sample size (*n*), considering the design effect (*Deff*) and the actual sample size (*n*′), was calculated as follows:

$$n=n^{\prime }/Deff$$

Secondly, the margin of error (*e*) for the various WO was calculated for each individual year based on the following equation:

$$n=\frac{Z^{2}\;\left(\left(Z\alpha /2+Z\beta \right)2\sigma^{2}\right)}{e^{2}\;}$$

where *n* is the sample size, *σ*^2^ is the variance of the WO, and Z is the value from a standard normal distribution, corresponding to the desired confidence level as the type I error Alpha (Z = 1.96 for 95 percent confidence interval (CI)), and Beta the power of the analysis set at 80%, which is considered acceptable. Finally, we assessed if the margins of error overlapped between years in order to estimate the confidence in the trend identified over the years for the different WO.

### 2.6. Software

Data processing and data analyses were carried out using Microsoft Excel Office Professional Plus 2010 (Microsoft, Redmond, Washington, WA, USA) and RStudio (4.0.3 for Window 64 bit, Boston, MA, USA). The lme4, mvtnorm and multcomp R Packages were used for the glmm analysis, Tukey’s HSD and cld. The forecast, tseries and ggplot2 R packages were used for the time series analyses.

## 3. Results

### 3.1. Number of Pigs, Pens and Farms Visited

The farm visits were repeated with a minimum of 1 visit per year and a maximum of 6 visits per year, dependent on scheme entry date and the presence of finishing pigs on the farm, so these farms were visited between one and maximum 29 times since the “Real Welfare” scheme was first implemented. In total, 7.8% of the farms had only one visit which occurred between 4 April 2013 and 30 December 2019. Moreover, 5.0% of the farms had only one visit which occurred at least one year ago (before 1 January 2019), suggesting that these farms might no longer be part of the “Real Welfare” scheme. It is likely that many of these were finishing pig sites temporarily contracted by integrators. The rest of the farms with one visit might have joined the scheme recently and completed only one visit by the end of 2019. In total, 20,240 farm visits have been conducted in 2616 different farms.

The average number of finishing pig places was 1583 pigs per farm and the average number of finishing pigs present in the farm at the time of assessment was 1213 pigs per farm. Ninety percent of the farms had 400 to 3060 pig places in the farm. Based on the number of pig places, the average percentage of pigs assessed per visit was 23.0% for severe lesions and 44.6% for hospitalization requirement or lameness. In total, 0.1% of the farms had missing values for the percentage of pigs assessed. The number of pens selected largely depended on the farming system and the herd size. The average number of pigs per pens was 56. On average, 9.6 pens per farm were assessed per visit, with a minimum of one and a maximum of 98 pens. On average, 2.2 buildings were visited per farm and per visit with a minimum of 1 and a maximum of 13. The number of farms, pens and pigs assessed for each lesion (including the mild lesions and enrichment use ratio) in each year of the “Real Welfare” scheme is documented in Supplementary Table S1. Approximately 2,000,000 pigs were assessed per year for mandatory WO.

### 3.2. Association between the Different Welfare Outcomes and Overall Prevalence

**Figure 2:**
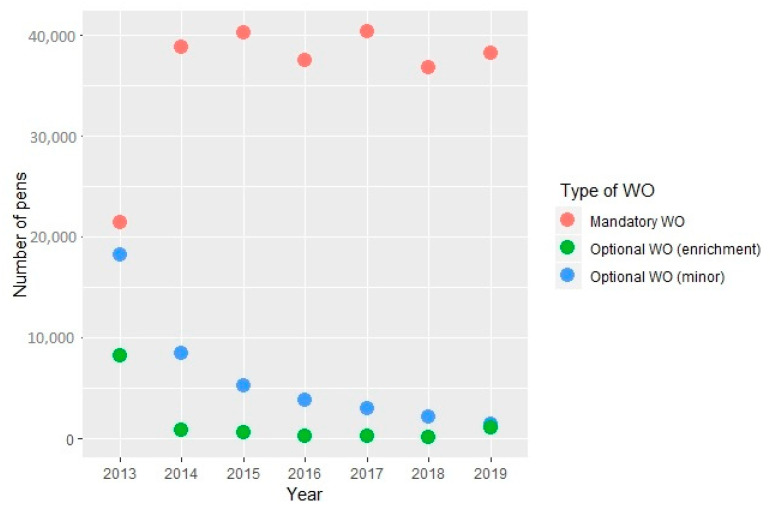
Number of pens assessed per year for the mandatory welfare outcome (WO) (pigs requiring hospitalization, lame pigs, severe tail lesions, severe body marks) and optional welfare outcomes (WO) (minor: mild tail lesions and dirty tail, mild body marks and dirty body; enrichment: enrichment use ratio) for the data collected between 4 April 2013 and 31 December 2019.

The mean percentage of each WO was calculated at the pig level (Table 1), the pen level (Table 2) and the farm level (based on annual rolling average) (Table 3). While the number of pens assessed per year increased in 2014 and remained stable in the subsequent years for the mandatory WO, the number of pens assessed per year constantly decreased for the optional WO (minor lesions and enrichment use ratio) (Figure 2). The only significant correlation among the mandatory WO was between the percentage of pigs requiring hospitalization and the percentage of pigs with lameness (*p* < 0.05, *r* = 0.34). When only the farms with minor lesions recorded were considered, the correlation between the percentage of pigs requiring hospitalization and the percentage of pigs with lameness remained unchanged, and the percentage of dirty tail was moderately correlated to the percentage of dirty body (*p* < 0.05, *r* = 0.49). No significant correlations were identified between minor and severe lesions (*r* < 0.30).

### 3.3. Assessment of the Changes over the Years, Visits and Seasons of the Different Welfare Outcomes

#### 3.3.1. Change over the Years

**Figure 3:**
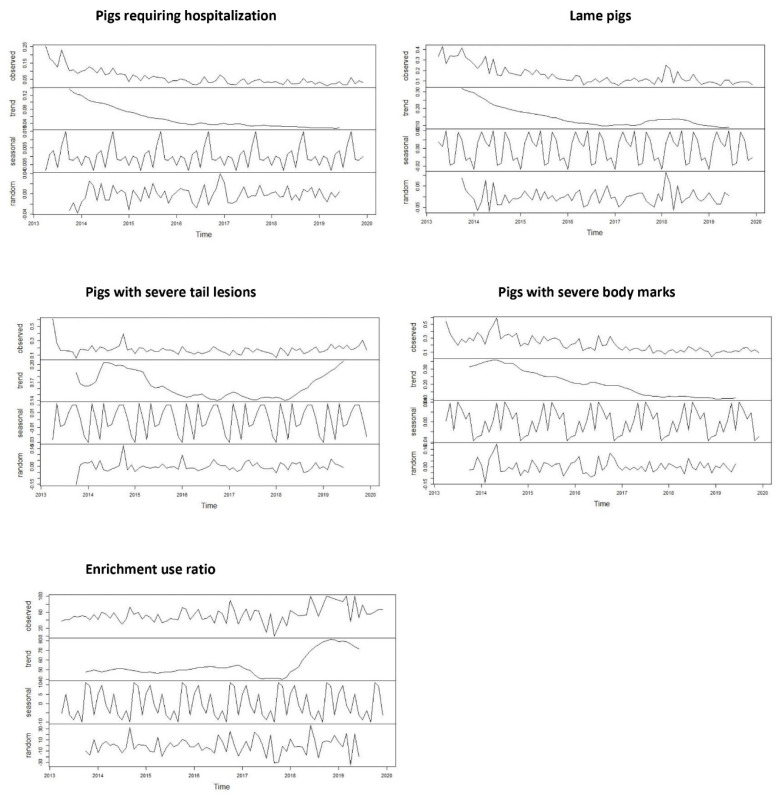
Decomposition of the time series of the percentage of pigs requiring hospitalization, lame pigs, pigs with severe tail lesions, severe body marks and enrichment use ratio from April 2013 to December 2019 (observed = gross pattern, trend = underlying trend, seasonal = seasonal component, and random = residual variations).

Following the time series analyses, “seasonal” patterns were identified for all WO (Figure 3). For all WO, the *p*-value of the ADF test was greater than 0.05, indicating non-stationary time series and inconsistent change over time. For the time series of pigs requiring hospitalization, lame pigs and pigs with severe body marks, we could identify a slow and irregular decrease in the ACF as the lags increased; this is due to the trend and the seasonality observed in these time series (Supplementary Figure S1). We observed a decreasing trend in the percentages of pigs requiring hospitalization, lame pigs, and severe body marks, but not for the percentage with severe tail lesions and the enrichment use ratio (Figure 3).

Firstly, using generalized linear mixed models, all subsequent years were compared to 2013 (the first year of the “Real Welfare” scheme). Secondly, a pair-wise comparison between years, based on Tukey’s HSD method and cld function, was used to identify sets of years that were not detectably different from each other. Finally, the results were used to conclude changes in prevalence over time of these WO.

The proportion of pigs requiring hospitalization was significantly lower in 2019 and 2018 compared to 2015, 2014 and 2013 (*p* < 0.001) (Table 4, Supplementary Tables S2 and S4). The proportion of pigs requiring hospitalization was significantly lower in 2019 compared to 2017 and 2016 (*p* < 0.005). This suggests a smaller mean prevalence in most recent years compared to the first years of the period covered by the scheme.

The proportion of lame pigs was significantly lower in 2019 compared to 2016 (*p* < 0.05), 2018, 2015, 2014 and 2013 (*p* < 0.001). The proportion of lame pigs was significantly lower in 2018 compared to 2015, 2014 and 2013 (*p* < 0.001) (Table 4, Supplementary Tables S2 and S4). This suggests a smaller mean prevalence in most recent years compared to the first years of the period covered by the scheme.

The proportion of pigs with severe tail lesions was significantly higher in 2019 compared to 2013, 2015, 2016, 2017, and 2018 but was not significantly higher than 2014. The proportion of pigs with severe tail lesions was significantly higher in 2018 compared to 2017 and 2013 but not the other years (Table 4, Supplementary Tables S2 and S4). This suggests no significant differences in the mean prevalence between the first years and last years of the period covered by the scheme.

The proportion of pigs with severe body marks was significantly higher in 2013, 2014, 2015, and 2016 compared to 2018 and 2019 and significantly higher in 2017 compared to 2019 (*p* < 0.001) (Table 4, Supplementary Tables S2 and S4). This suggests a smaller mean prevalence in most recent years compared to the first years of the period covered by the scheme.

The enrichment use ratio was significantly lower in 2013, 2014, 2015, and 2016 compared to 2018 and 2019 and significantly lower in 2013, 2014 compared to 2015, 2016, 2017, 2018, 2019 (*p* < 0.001) (Table 4, Supplementary Tables S2 and S4). This may suggest an increasing trend which appears mainly over the two last years. However, the diminishing sample size once this outcome became optional must be borne in mind.

For the WO with optional recording, the results are summarized in Table 5 and Supplementary Tables S3 and S5 but would need to be confirmed by further analyses with mandatory recording. The results suggest an initial decline of mild tail lesions between 2013 and 2014, which cannot be interpreted as the recording of mild lesion became optional in 2014. However, there is a further decline between 2014 and 2016 which tends to flatten out in the subsequent years. The results also suggest a progressive increase in the proportion of pigs with dirty tails between 2014 and 2019, a significant decline each year of the proportion of pigs with mild body marks, and an increase between 2017 and 2019 of the proportion of pigs with dirty bodies.

#### 3.3.2. Change over Calendar Seasons

The results of the generalized linear models suggest differences between calendar seasons in the pigs requiring hospitalization, lame pigs, pigs with severe tail lesions, pigs with severe body marks and enrichment use ratio (Table 6, Supplementary Table S6). The different outcomes did not show a similar seasonal pattern. The proportion of pigs requiring hospitalization was significantly higher in autumn and winter compared to spring (*p* < 0.001, *p* = 0.006). The proportion of lame pigs was significantly higher in winter compared to summer. The proportion of pigs with severe tail lesions was significantly higher in spring and autumn compared to summer and winter (*p* < 0.001). The proportion of pigs with severe body marks was significantly lower in autumn compared to spring (*p* < 0.001). The enrichment use ratio was significantly higher in winter compared to summer, autumn and spring (*p* < 0.001). The enrichment use ratio was significantly higher in autumn compared to spring (*p* < 0.001) (Supplementary Table S7).

#### 3.3.3. Changes over the Sequential Assessment Visits

**Figure 4:**
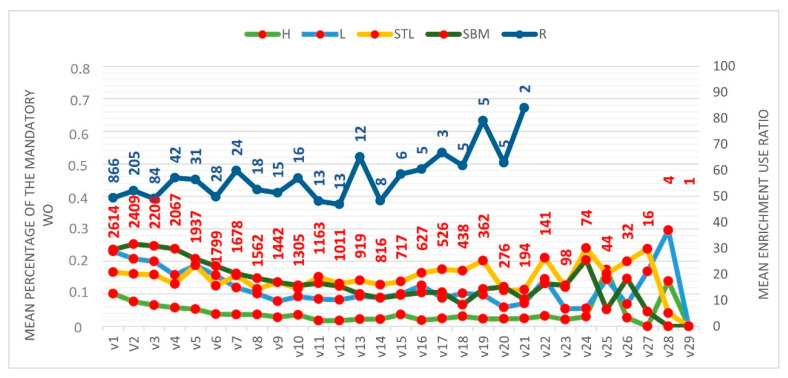
Mean percentage of the mandatory welfare outcomes (WO) (Pigs requiring hospitalization (H), lame pigs (L), pigs with severe tail lesions (STL), pigs with severe body marks (SBM) and enrichment use ratio (R) from the 1st to the 29th visit (21st visit for enrichment use ratio). The number of farms included in the sample to calculate the mean for each visit is reported on the graph in red for the mandatory welfare outcomes and in blue for the enrichment use ratio.

The general trend within farm over successive visits is visualized in Figure 4. The percentages for the last visits cannot be interpreted, as very few farms were included in the sample. While a decreasing trend can be observed over the visits for the mean percentage of pigs requiring hospitalization, lame pigs and pigs with severe body marks, the other WO remained more stable.

There was no Kendall tau-b correlation between the different visits for the percentage of pigs requiring hospitalization, confirming changes over the visits for this WO with an overall downward trend. Moderate correlations were identified for the percentage of lame pigs, mainly between consecutive visits. The correlations were weaker for visits with wider interval, suggesting that changes occurred but could be slower compared to the percentage of pigs requiring hospitalization. Indeed, a downward trend over visits was also identified. The Kendall’s tau-b correlation showed no correlation between the different visits for the percentage of pigs with severe tail lesions confirming changes over the visits. However, looking at the graph summarizing the mean WO prevalence per visit, no trend could be identified. Indeed, the mean percentage of pigs with severe tail lesions tend to go up and down around a mean value, suggesting that some farms experienced an increase in this WO while others experienced the opposite. Moderate correlations for the percentage of pigs showing severe body marks, mainly between consecutive visits, indicated small changes between these visits. Moreover, the lack of correlation between other visits with wider intervals suggests that changes occurred but could be slower compared to the percentage of pigs requiring hospitalization. Indeed, a downward trend over visits was also identified. The correlation between several visits, even with a longer interval between visits, suggests only moderate changes in the enrichment use ratio over the visits. No specific trend could be observed in the graph summarizing the mean enrichment use ratio per visit (Figure 4) (Supplementary Table S8).

### 3.4. Precision of the Estimates

**Figure 5:**
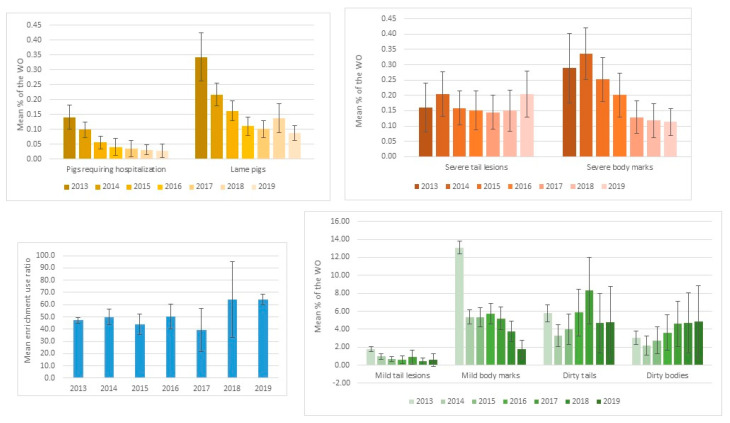
Annual mean and margin of error considering the sampling size (accounting for the design effect) and 80% power for all welfare outcomes (WO).

Considering the sampling size, we can see that the margin of error is not always overlapping for pigs requiring hospitalization, lameness, severe body marks, mild body marks and enrichment use ratio (Figure 5) (Supplementary Table S9). This indicates that the sampling size allows good confidence in the trend observed over the years, keeping in mind that the recording of enrichment use ratio and mild body marks was not mandatory.

## 4. Discussion

### 4.1. Study Limitations

Despite a very low prevalence of some measures, the large amount of data available from the “Real Welfare” scheme allows more accurate analysis compared to studies based on primary data, where data are often collected on a restricted number of farms. However, absence of data related to hospital pens makes it difficult to separate a real decrease in the prevalence of the different WO over time from a better management of compromised pigs. Since the WO were only recorded in the mainstream herd, the decreasing trend in several WO could be explained by either a real reduction in these WO or better management of transfer to hospital pens or euthanasia of the most severely injured pigs, but both would contribute to overall improvement in pig herd welfare. The possible change in management of compromised pigs could be due to the impact of the vet assessor on farmer perception of sick pigs, whereby regular visits of the vet operating welfare assessment might have changed the perception of the farmer regarding sick pigs and hospital pen management [13]. Another bias, due to repeating assessments over time and the absence of “update training” of the vets might have been an impact on inter- and intra-observer reliability. However, several studies have shown good inter-observer reliability of similar WO data recorded by trained assessors [14,15]. We also included the interaction of the vet with the farm in the models that assessed the changes over time and season in order to reduce any information bias in our analysis. Regarding the additional time series analysis, which confirms the decreasing trend of some WO, the autoregressive model used in this analysis might appear too simplistic. While this model considers its previous terms as the predictors, there is likely more complexity than a simple linear model of past observation and a more complex model should be used to create forecasts.

Using a large sample size and conducting an assessment of pigs randomly selected at the pen level appear to be the best options to evaluate WO [16]. Indeed, high variability can exist between pens of the same farm, which makes it difficult to accurately assess farm prevalence with reduced sample size [17]. Moreover, assessing the prevalence of the different WO at the pen level appeared to be more informative in another study [18]. The sample of pig farms assessed across each year by the “Real Welfare” scheme produces approximately 95% of all commercially raised pigs in the UK and, for each visit, the assessment was conducted on a representative sample of pigs and pens selected by trained vets [11]. This allowed us to conduct the analysis on a large sample representative of the mainstream herd of finishing pigs in the UK. Following the analysis of the margin of error for each individual year, considering 80% power, we could conclude that the sampling allows accurate estimation of the prevalence of these different WO and allows accurate assessment of the changes in prevalence over time. However, the recording of enrichment use ratio and minor lesions was not mandatory after 2013 and this may have led to selection bias and lower precision in the estimates. The results should therefore be interpreted with caution because the number of pens which were recorded for these lesions drastically dropped in recent years. For the same reason, the trend over visits (using visualization of the graph in Figure 4 and Kendall’s tau-b correlation) was not interpreted beyond eight visits because of the declining sample size. However, we decided to share the results on enrichment use ratio and minor lesions because the sample remains exceptionally large compared to most other studies. Moreover, highlighting the decreasing number of pens assessed over time (when the measures became optional) in itself constitutes interesting information for the pig sector and future projects. Pen selection, pen size and the variability between pens will impact the precision of the estimate (margin of error) [19,20]. In the “Real Welfare” scheme, only a subset of the pens and subset of the pigs according to farm size were sampled in order to be representative of pigs present in the farm, but also to reduce time and commitment required on the operational aspects [9]. To get exactly the same level of precision in each farm, a specific sampling calculation which considers farm design could be applied in each individual farm but, at a large scale, this calculation would represent a constraint that could impair the simplicity of implementation of the assessment.

### 4.2. Prevalence of the Welfare Outcomes

The mean prevalence of the mandatory WO was very low at the pig, pen and farm levels. Similar results regarding the prevalence of the different WO were obtained in previous studies [21,22,23]. The standard deviation was higher than the mean for all WO, indicating that the percentage of WO in the different pens and farms spreads out over a wide range of values. The percentage of pigs with a welfare issue could be very high in certain individual pens, but the mean percentage of pigs with these welfare issues at the farm level was much lower. This confirms that these welfare issues remain rare and sporadic [18,22]. A previous paper, based on the data recorded between 2013 and 2016, identified several risk factors associated to higher prevalence of the different WO [10]. However, more detailed analysis could be done on farms and pens with high prevalence of WO to better understand the combination of factors that lead to welfare issues and identify other risk factors. Furthermore, such high prevalence cases should be the subject of more detailed investigation and remedial action by the farmer and veterinarian whenever they are observed.

Based on the European Food Safety Authority (EFSA) [24] report, the prevalence of tail lesions on farm may vary widely (of the order of 1–5%). In our study, the percentage of pigs with severe tail lesions and severe body marks was 0.04% and 0.12%, respectively. Some studies which evaluated the prevalence of tail damage reported a prevalence of severe tail biting at the pig level of 1.3% in Finland [25] and 2.2% in France (0.9% for the most severe lesions) [7]. The mean percentage at the batch level from farrow-to-finish herds (representing 12% of the Irish herd) was 1.2% for severe tail lesions and 1.2% severe skin lesions [8]. Comparisons are difficult to make because the precise definition of a lesion differs between studies. A similar sampling methodology and classification should be adopted to enable valid comparison between studies.

A higher percentage of pigs requiring hospitalization was associated with a higher percentage of lame pigs. This may be due to similar risk factors within a pen for both of these welfare issues, or to the fact that individual pigs may fall into both categories (lameness could be a common reason for moving pigs in hospital pens). Some correlation between these two WO was also identified in a previous study [26]. Different results were found by Munsterhjelm et al. [27], who also excluded hospital pens from their analysis and found a connection between wounds and lameness. However, both samples were much smaller in these studies. The absence of other correlations between WO suggests that all WO are specific and complementary in welfare assessment and, therefore, could reveal different aspects of animal welfare. Indeed, it has been shown that pigs in hospital pens are associated with both respiratory and locomotion problems [16], lame pigs are associated with both flooring and the detection of *Mycoplasma hyosynoviae* [28], and body and tail lesions are associated with various stressors and negative social behavior [29,30]; all acting as signals of impaired animal well-being.

### 4.3. Changes over Time and Season of the Welfare Outcomes

Following our analysis, we could conclude that pigs requiring hospitalization, lame pigs, severe body marks and mild body marks decreased over the years and enrichment use ratio increased over time. No trend could be identified for severe tail lesions, as similar prevalence was observed in 2014 and 2019. Dirty tails and dirty body tended to increase in recent years covered by the “Real Welfare” scheme, though the sample size became very small and the trend observed should be interpreted with care. Considering the very low percentage prevalence of all WO in the most recent years, we could easily expect a stabilization of the prevalence, as a complete absence of welfare issues seems to be unrealistic. While the “Real Welfare” scheme might be considered to have provided good support to decrease lameness, severe body marks or improve hospital pen management, severe tail lesions may be greatly influenced by other less controllable factors, such as the housing infrastructure of the farm, diet formulation or climatic variation, making changes over time less visible. The identification by the farmer of the different farm-specific risk factors is essential. Tools such as “WebHAT” or “SchwIP” have been created to raise awareness amongst farmers about the risk factors and may offer a better help to reduce tail biting [21,31]. It is known that benchmarking of health and welfare measures can lead to greater awareness and motivation to improve [32] but our study suggests that this is not uniformly successful for all WO, especially for severe tail lesions. While other welfare assessment initiatives exist and measure similar WO, such a large-scale assessment as reported here is not documented in the literature for other countries. The implementation of a similar scheme in other countries would allow comparisons of the prevalence and the trend of the different WO between countries.

While a decline over repeated assessment visits to individual farms could be clearly seen for the percentage of pigs requiring hospitalization, the percentage of lameness and severe body marks, no trend over visits was identified for the percentage of pigs with severe tail lesions and enrichment use ratio. Only the percentage of pigs requiring hospitalization showed a decreasing trend with no correlation between years, suggesting quicker changes compared to the percentage of lame pigs and the percentage of severe body marks, which showed correlations between consecutive visits. No correlations between visits were identified for severe tail lesions, suggesting both increases and decreases according to the farm, without identifying a general trend. In contrast, the enrichment use ratio showed significant correlations between visits, suggesting no particular changes over visits.

‘Seasonal’ changes identified in the time series analysis are difficult to interpret. Indeed, seasonal changes probably act as a proxy for other factors related to environmental or management differences or may be connected to other seasonal health issues. For example, some health issues have been reported to be more common during winter [33,34]. Severe tail lesions were more prevalent in autumn and spring, corresponding to times with greater temperature fluctuations. Higher enrichment use ratio was observed in autumn and winter, possibly associated with greater bedding supply at these times.

## 5. Conclusions

The estimate of the mean percentage of the different WO in the mainstream herd of the UK finishing pig population was ≤0.2% when assessed at the pig, pen and farm levels, but a high prevalence was detected sporadically. In the period since the implementation of the “Real Welfare” scheme, our analysis indicates an improvement in welfare in the mainstream herd of finishing pig farms in the UK, reflected in the reduction in the prevalence of the main WO (except for severe tail lesions) over both calendar years and sequential assessment visits at a given farm. The decreasing trend over both years and successive assessment visits tended to flatten out, suggesting that further decline from the present low values would be difficult to achieve. The “Real Welfare” scheme might have contributed to a decrease in the prevalence of certain WO in the mainstream herd by stimulating a reduction in causal factors for these welfare problems and/or an improvement in the management of compromised pigs. The possible influence of the scheme should be confirmed by further studies. Other initiatives, such as increasing farmer awareness, identification and understanding of risk factors for tail biting and other outcomes, and support for changes in farm management should also be considered.

## Figures and Tables

**Table 1:** Description of the welfare outcomes at the pig level for all pigs assessed between 2013 and 2019: mean percentage and 95% confidence interval [CI].

Welfare Outcomes	Mean [CI] ^2^
Pigs requiring hospitalization	0.04 [0.04–0.04]
Lameness	0.12 [0.12–0.12]
Severe tail lesions	0.15 [0.15–0.16]
Severe body marks	0.18 [0.17–0.18]
Mild tail lesions ^1^	1.07 [1.05–1.09]
Mild body marks ^1^	8.55 [8.49–8.60]
Dirty tail ^1^	5.82 [5.77–5.86]
Dirty body ^1^	3.96 [3.92–3.99]
Enrichment use ratio	54.4 [54.1–54.7]

^1^ Includes only the pens where minor lesions were assessed; ^2^ the value is the percentage of pigs exhibiting each outcome among the pigs sampled, or the percentage of pigs directing their exploratory behavior to the enrichment item provided among pigs sampled directing their exploratory behavior toward enrichment item provided or other pen features or pen mates.

**Table 2:** Description of the welfare outcomes at the pen level for all pens visited between 2013 and 2019: Mean percentage, standard deviation (SD), minimum (MIN) and maximum (MAX) values, median, 10% percentile (P10), and 90% percentile (P90).

Welfare Outcomes	Mean [CI] ^3^	SD	Min	P10	Median	P90	Max	Min ^2^	Median ^2^	Max ^2^
Pigs requiring hospitalization	0.06 [0.06–0.06]	0.72	0	0	0	0	100	0	0	50
Lameness	0.15 [0.15–0.16]	1.11	0	0	0	0	100	0	0	66.7
Severe tail lesions	0.17 [0.16–0.18]	1.67	0	0	0	0	100	0	0	100
Mild tail lesions ^1^	1.25 [1.12–1.29]	4.80	0	0	0	4	100	0	0	100
Dirty tail ^1^	5.40 [5.35–5.56]	16.2	0	0	0	16.7	100	0	0	100
Severe body marks	0.20 [0.19–0.21]	1.62	0	0	0	0	100	0	0	100
Mild body marks ^1^	9.55 [9.41–9.69]	14.7	0	0	4	27.8	100	0	4	100
Dirty body ^1^	3.53 [3.39–3.66]	13.6	0	0	0	5	100	0	0	100
Enrichment use ratio	48.9 [48.2–49.5]	35.6	0	0	50	100	100	0	50	100

^1^ Includes only the pens where mild lesions were assessed; ^2^ values based on annual rolling averages; ^3^ the value is the percentage of pigs in the pen exhibiting each outcome among the pigs sampled, or the percentage of pigs directing their exploratory behavior to the enrichment item provided among pigs sampled directing their exploratory behavior toward enrichment item provided or other pen features or pen mates.

**Table 3:** Description of the welfare outcomes at the farm level (% of pigs or ratio) for all farms visited between 2013 and 2019: mean percentage and 95% confidence interval [CI], standard deviation (SD), minimum (MIN) and maximum (MAX) values, median, 10% percentile (P10), and 90% percentile (P90).

Welfare Outcomes ^2^	Mean [CI] ^3^	SD	Min	P10	Median	P90	Max
Pigs requiring hospitalization	0.05 [0.05–0.05]	0.18	0	0	0	0	5.11
Lameness	0.15 [0.15–0.16]	0.47	0	0	0	0	18.7
Severe tail lesions	0.15 [0.14–0.16]	0.60	0	0	0	0	23.9
Mild tail lesions ^1^	1.12 [1.03–1.22]	2.25	0	0	0.18	4	29.2
Dirty tail ^1^	6.98 [6.32–7.64]	16.2	0	0	0	16.7	100
Severe body marks	0.18 [0.17–0.20]	0.68	0	0	0	0	14.6
Mild body marks ^1^	9.68 [9.20–10.2]	11.6	0	0	5.65	27.8	82.7
Dirty body ^1^	5.06 [4.47–5.64]	14.0	0	0	0	5	97.7
Enrichment use ratio	51.7 [51.2–52.2]	25.9	0	0	52.5	100	100

^1^ Includes only the pens where mild lesions were assessed; ^2^ values based on annual rolling averages; ^3^ the value is the percentage of pigs in the pen exhibiting each outcome among the pigs sampled, or the percentage of pigs directing their exploratory behavior to the enrichment item provided among pigs sampled directing their exploratory behavior toward enrichment item provided or other pen features or pen mates.

**Table 4:** Mean percentage and 95% confidence interval [CI] for each year (without considering random effect) and Compact Letter Display (cld) for mandatory welfare outcomes and enrichment use ratio. Values with a different cld are significantly different (*p* < 0.05).

	Pigs Requiring Hospitalization		Lame Pigs		Severe Tail Lesions		Severe Body Marks		Enrichment Use Ratio	
Year	Mean [CI]	cld	Mean [CI]	cld	Mean [CI]	cld	Mean [CI]	cld	Mean [CI]	cld
2013	0.14 [0.13–0.15]	e	0.34 [0.32–0.37]	e	0.16 [0.14–0.18]	A	0.29 [0.26–0.32]	e	47.1 [46.6–47.6]	a
2014	0.10 [0.09–0.11]	d	0.22 [0.20–0.23]	d	0.20 [0.18–0.22]	Bcd	0.34 [0.31–0.36]	e	49.8 [49.5–50.2]	a
2015	0.06 [0.05–0.06]	c	0.16 [0.15–0.17]	c	0.16 [0.14–0.17]	bc	0.25 [0.23–0.27]	d	43.9 [43.6–44.3]	b
2016	0.04 [0.03–0.05]	b	0.11 [0.10–0.12]	b	0.15 [0.13–0.17]	ac	0.20 [0.18–0.22]	c	50.3 [49.9–50.6]	b
2017	0.03 [0.03–0.04]	b	0.10 [0.09–0.11]	ab	0.14 [0.13–0.16]	ab	0.13 [0.12–0.14]	a	39.4 [39.1–39.7]	ab
2018	0.03 [0.03–0.04]	ab	0.14 [0.12–0.15]	bc	0.15 [0.13–0.17]	c	0.12 [0.11–0.13]	ab	64.1 [63.8–64.4]	c
2019	0.03 [0.02–0.03]	a	0.09 [0.08–0.10]	a	0.20 [0.19–0.22]	d	0.11 [0.10–0.12]	b	64.1 [63.8–64.4]	c

**Table 5:** Mean percentage and 95% confidence interval [CI] for each year (without considering random effect) and Compact Letter Display (cld) for optional welfare outcomes. Values with a different cld are significantly different (*p* < 0.05).

	Mild Tail Lesions		Dirty Tails		Mild Body Marks		Dirty Bodies	
Year	Mean [CI]	cld	Mean [CI]	cld	Mean [CI]	cld	Mean [CI]	cld
2013	1.76 [1.68–1.83]	e	5.77 [5.55–6.00]	abc	13.1 [12.9–13.3]	g	3.06 [2.88–3.23]	abc
2014	0.98 [0.89–1.06]	d	3.27 [2.99–3.55]	a	5.36 [5.14–5.59]	f	2.17 [1.93–2.40]	ab
2015	0.68 [0.59–0.77]	c	3.99 [3.61–4.37]	a	5.35 [5.06–5.63]	e	2.76 [2.42–3.10]	a
2016	0.61 [0.48–0.74]	ab	5.85 [5.27–6.44]	b	5.74 [5.40–6.09]	d	3.61 [3.17–4.06]	b
2017	0.87 [0.66–1.09]	bcd	8.31 [7.48–9.14]	b	5.19 [4.83–5.55]	c	4.59 [4.03–5.16]	c
2018	0.42 [0.32–0.53]	a	4.68 [3.94–5.42]	c	3.74 [3.41–4.06]	b	4.68 [3.93–5.43]	d
2019	0.56 [0.36–0.76]	a	4.77 [3.88–5.65]	c	1.74 [1.46–2.03]	a	4.82 [3.93–5.71]	d

**Table 6:** Mean percentage and 95% confidence interval [CI] for each calendar season (without considering random effect) and Compact Letter Display (cld) for mandatory welfare outcomes and enrichment use ratio. Values with a different cld are significantly different (*p* < 0.05).

	Pigs Requiring Hospitalization		Lame Pigs		Severe Tail Lesions		Severe Body Marks		Enrichment Use Ratio	
Season	Mean [CI]	cld	Mean [CI]	cld	Mean [CI]	cld	Mean [CI]	cld	Mean [CI]	cld
Spring	0.11 [0.09–0.13]	a	0.38 [0.34–0.42]	ab	0.20 [0.16–0.25]	b	0.23 [0.18–0.27]	a	41.6 [40.9–42.4]	b
Summer	0.13 [0.11–0.15]	ab	0.30 [0.28–0.33]	a	0.15 [0.13–0.17]	a	0.19 [0.16–0.22]	ab	47.5 [46.9–48.1]	bc
Autumn	0.12 [0.10–0.13]	b	0.36 [0.33–0.39]	ab	0.16 [0.12–0.19]	b	0.21 [0.19–0.24]	b	49.3 [48.7–49.9]	c
Winter	0.09 [0.07–0.11]	b	0.30 [0.25–0.34]	b	0.16 [0.12–0.20]	a	0.18 [0.13–0.22]	ab	54.8 [54.1–55.6]	a

## Data Availability

The datasets generated and analyzed during the current study are not publicly available as the ownership of the non-anonymized data is that of each individual farmer.

## Supplementary Materials

The following supporting information can be downloaded at: https://www.mdpi.com/article/10.3390/ani12050607/s1. Material S1; Methods S1; Figure S1: Autocorrelation function (ACF) (with lag= fixed amount of passing time) for the percentage of pigs requiring hospitalization, lame pigs from, pigs with severe tail lesions, severe body marks and enrichment use ratio April 2013 to December 2019; Table S1: The number of farms, pens, pen visits and pigs assessed for each lesion (including the mild lesions and enrichment use ratio) in each year of the scheme (For the calculations for each year, pens without a visit date have been excluded), Table S2: Changes over years compared to 2013 for the different welfare outcomes: Odds ratio, confidence intervals and P-value for all pens included in the study, Table S3: Changes over years compared to 2013 for the minor welfare outcomes: Odds ratio, confidence intervals and P-value for all pens with minor lesions recorded (42,108 pens), Table S4: *p*-values for pair-wise comparison between years with Tukey’s HSD for the proportion of pigs requiring hospitalization, lame pigs, pigs with severe tail lesions, pigs with severe body marks and enrichment use ratio, Table S5: *p*-values for pair-wise comparison between years with Tukey’s HSD for the proportion of pigs with mild tail lesions, dirty tails, mild body marks, dirty bodies, Table S6: Changes over seasons compared to winter for the mandatory welfare outcomes and enrichment use ratio: Odds ratio, confidence intervals and P-value for all pens included in the study, Table S7: values for pair-wise comparison between seasons with Tukey’s HSD for the proportion of pigs requiring hospitalization, lame pigs, pigs with severe tail lesions, pigs with severe body marks and enrichment use ratio, Table S8: Kendall’s tau-b correlation coefficient between the average percentages of welfare outcomes (H: pigs requiring hospitalization, L: lame pigs, STL: pigs with severe tail lesions, SBM; pigs with severe body marks, R: enrichment use ratio) for individual farms in each visit (1st to 8th visits). (*p* < 0.05, Values > 0.3 represent a significant correlation), Table S9: Annual mean and calculated margin of error considering the sampling size of each year (accounting for the design effect) and 80% power for all welfare outcomes.
