# Supplementary material for: The “Real Welfare” Scheme: Changes in UK Finishing Pig Welfare since the Introduction of Formal Welfare Outcome Assessment
Source: Animals (Basel). 2022 Feb 28;12(5):607. doi: 10.3390/ani12050607 (PMC8909812; doi:10.3390/ani12050607)
Supplement: Supplementary file 1 [file animals-12-00607-s001.zip › animals-1586305-supplementary.pdf]

Supplementary file

Supplementary Material S1 Sampling and data collection.

The number of assessments per farm per year (2 to 4) depended on pig flow and was achieved during a quarterly visit from the farm veterinarian. All participating veterinarians were required to be members of the UK Pig Veterinary Society and were required to undergo online and practical training to ensure standardisation of recording [http://pork.ahdb.org.uk/health-welfare/welfare/real-welfare/real-welfare-vets/]. The assessment involved 5 main measures. Full details of the measurement protocol can be found at [http://pork.ahdb.org.uk/health-welfare/welfare/real-welfare/]. Tail lesions and body marks were assessed on a sample of pigs per pen, but pigs requiring hospitalization, lame pigs and enrichment use ratio were assessed for all pigs in the selected pens as this method improved the accuracy of the recording of these welfare outcomes which usually occur at low prevalence. The number of pens assessed at each visit were selected to be representative of the farm and to comply with the number of pigs required to be assessed each year for tail lesions and body marks. For units of 300 finisher places or less, a minimum of 300 pigs should be sampled each year, but for units of 900 finisher places or more, a total of 900 pigs should be sampled per year. For units of between 300 to 900 finisher places, an equivalent representative proportion should be sampled. The sampling of pigs within a pen was as follows: all pigs in the pen if there were fewer than 25 pigs, 25 pigs if there were up to 100 pigs in the pen, or 50 pigs if there were more than 100 pigs in the pen. Sampling more pigs than this per pen was allowed at the vets' discretion and if the total number of pigs required to be sampled on farm could not otherwise be reached (for instance if a farm has only few pens, but with many pigs). In this case, the recommendation was to divide the number of pigs needed from a pen type by the number of pens available (e.g. if 150 pigs were needed from two pens of 100, sample 150/2 = 75 pigs per pen). Data were preferentially collected from pigs of ≥50kg liveweight, but if there were not enough pigs for the sample then pigs of ≥30kg liveweight were also included in the sample.

Supplementary Methods S1

The Augmented Dickey-Fuller Test (ADF) tests the null hypothesis that a unit root is present in a time series sample. The test evaluates the stationary of a time series [1].The Autocorrelation function (ACF) defines how data points in a time series are related, on average, to the preceding data points [1].
The Tukey's Honest Significant Difference (Tukey's HSD) method is used to test differences among sample means for significance. The Tukey's HSD tests all pairwise differences while controlling the probability of making one or more Type I errors [2].
Compact Letter Display (cld) is used to generate a compact letter display of all pairwise comparisons of estimated marginal means. When a P value exceeds alpha, then the two means have at least one letter in common [3].

References

1. Venables, W.N.; Ripley, B.D. *Modern Applied Statistics with S*, 4th ed.; Springer: New York, NY, USA, 2002.
2. Salkind, N.J. *Encyclopedia of Research Design*, SAGE Publications: Thousand Oaks, CA, USA, 2010.
3. Piepho, H.-P. An algorithm for a letter-based representation of all pairwise comparisons. *J. Comput. Graph. Stat.* **2004**, *13*, 456–466.

Supplementary Table S1 The number of farms, pens, pen visits and pigs assessed for each lesion (including the mild lesions and enrichment use ratio) in each year of the scheme (For the calculations for each year, pens without a visit date have been excluded).

	Pigs requiring hospitalization/ lame pigs	Severe lesions[4]	Mild lesions[4]	Enrichment use ratio[2]
Number of farms[1]	**2616**	**2616**	**1371**	**975**
2013	1202	1202	1135	872
2014	1555	1555	409	87
2015	1638	1638	210	35
2016	1641	1641	176	15
2017	1671	1671	127	12
2018	1526	1526	107	9
2019	1615	1615	64	77
Number of pens[1]	**65023**	**65023**	**26360**	**7088**
2013	14225	14225	12698	6102
2014	22022	22022	5782	650
2015	24527	24527	3877	418
2016	22098	22098	2715	136
2017	22517	22517	2063	116
2018	20426	20426	1483	66
2019	20642	20642	1050	843
Number of pen visits	**253713**	**253713**	**42108**	**11035**
2013	21445	21445	18169	8188
2014	38888	38888	8455	838
2015	40266	40266	5194	536
2016	37544	37544	3747	205
2017	40429	40429	2982	149
2018	36846	36846	2120	66
2019	38295	38295	1441	1053
Number of pigs	**13678660**	**7112777**	**1062755**	**89165** [3]
2013	928902	520221	440148	64038
2014	1916291	1045362	213212	7785
2015	2005744	1078437	129914	4526
2016	1991931	1043692	98619	1944
2017	2313357	1226426	85079	1201
2018	2236311	1097757	62139	890
2019	2286124	1101782	33644	8781

1 Considering farms and pens may have been visited several times per year

2 Only the pens for which at least one animal was interacting with enrichment or other features or pen mates were included as these were the only ones for which it was possible to calculate an enrichment use ratio

3 Only pigs which interacted with enrichment or other features or pen mates were included, as these were the only ones for which it was possible to calculate an enrichment use ratio

4 Severe lesions: Pigs for which severe tail lesions and/or severe body marks were assessed. Mild lesions: Pigs for which minor tail lesions and /or minor body marks were assessed.

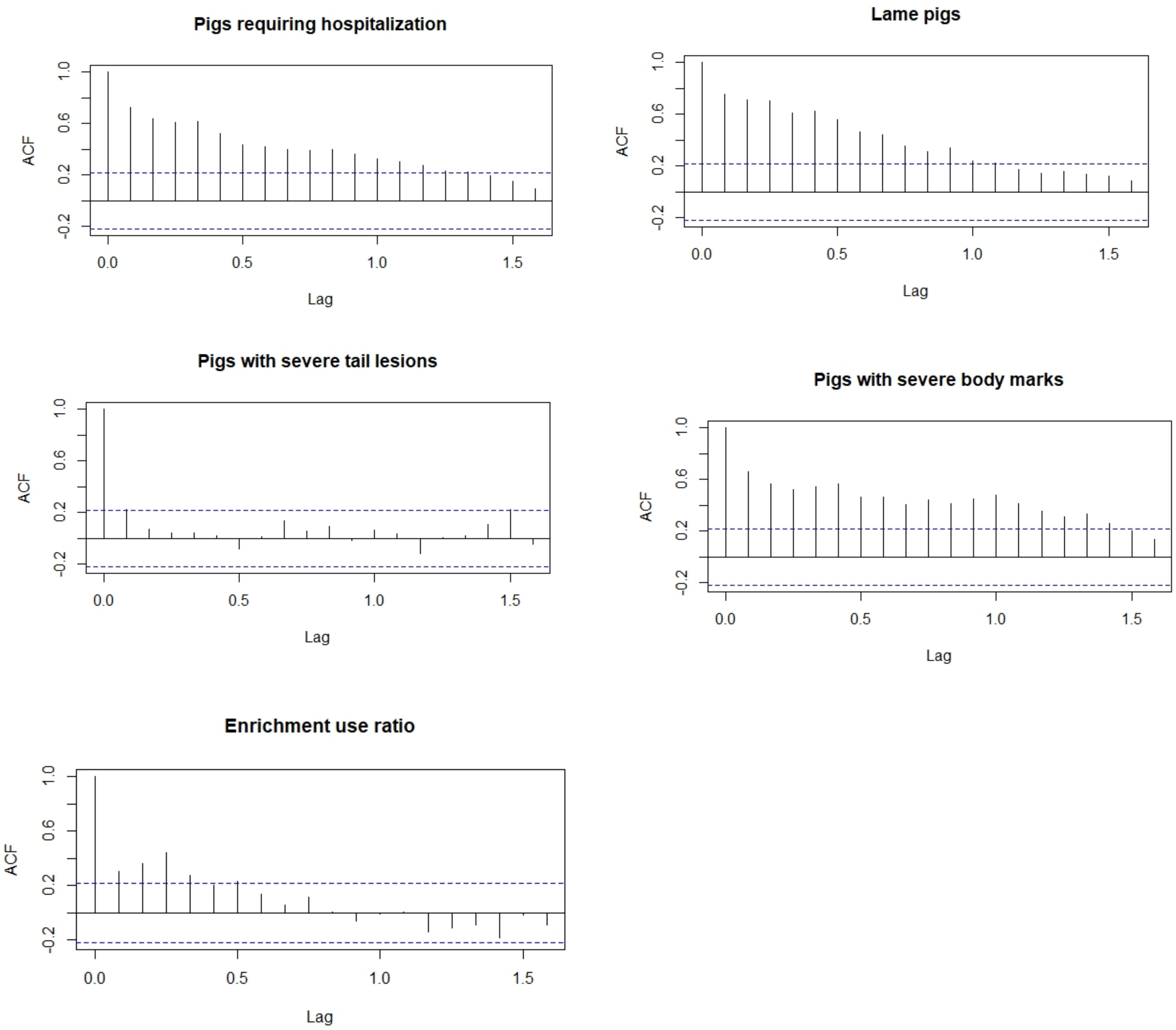

Supplementary Figure S1 Autocorrelation function (ACF) (with lag= fixed amount of passing time) for the percentage of pigs requiring hospitalization, lame pigs from, pigs with severe tail lesions, severe body marks and enrichment use ratio April 2013 to December 2019.

Supplementary Table S2 Changes over years compared to 2013 for the different welfare outcomes: Odds ratio, confidence intervals and P-value for all pens included in the study.

		Odd ratio	CI 95%		P-value
**Pigs requiring hospitalization**	2013	*Intercept*			
	2014	0.647	0.586	0.713	**< 0.001**
	2015	0.362	0.324	0.404	**< 0.001**
	2016	0.256	0.227	0.290	**< 0.001**
	2017	0.259	0.230	0.292	**< 0.001**
	2018	0.228	0.201	0.259	**< 0.001**
	2019	0.199	0.174	0.227	**< 0.001**
**Lameness**	2013	*Intercept*			
	2014	0.552	0.521	0.586	**< 0.001**
	2015	0.379	0.355	0.404	**< 0.001**
	2016	0.221	0.205	0.238	**< 0.001**
	2017	0.212	0.197	0.229	**< 0.001**
	2018	0.444	0.415	0.476	**< 0.001**
	2019	0.196	0.180	0.212	**< 0.001**
**Severe tail lesions**	2013	*Intercept*			
	2014	1.368	1.233	1.517	**< 0.001**
	2015	1.217	1.095	1.353	**< 0.001**
	2016	1.112	0.997	1.241	0.057
	2017	1.002	0.898	1.119	0.968
	2018	1.208	1.082	1.350	**0.001**
	2019	1.459	1.308	1.629	**< 0.001**
**Severe body marks**	2013	*Intercept*			
	2014	1.063	0.992	1.139	0.084
	2015	0.827	0.769	0.889	**< 0.001**
	2016	0.563	0.520	0.609	**< 0.001**
	2017	0.349	0.321	0.381	**< 0.001**
	2018	0.327	0.299	0.359	**< 0.001**
	2019	0.303	0.276	0.333	**< 0.001**
**Enrichment use ratio**	2013	*Intercept*			
	2014	1.018	0.930	1.114	0.697
	2015	1.101	1.013	1.198	**0.024**
	2016	1.520	1.372	1.685	**< 0.001**
	2017	1.542	1.351	1.759	**< 0.001**
	2018	1.154	0.986	1.351	0.075
	2019	2.656	2.198	3.210	**< 0.001**

Supplementary Table S3 Changes over years compared to 2013 for the minor welfare outcomes: Odds ratio, confidence intervals and P-value for all pens with minor lesions recorded (42,108 pens).

	Odd ratio	CI 95%		P-value
**Mild tail lesions**				
2013	*Intercept*			
2014	0.619	0.572	0.670	**< 0.001**
2015	0.535	0.482	0.595	**< 0.001**
2016	0.428	0.378	0.484	**< 0.001**
2017	0.793	0.703	0.895	**< 0.001**
2018	0.348	0.295	0.412	**< 0.001**
2019	0.358	0.291	0.439	**< 0.001**
**Dirty tails**				
2013	*Intercept*			
2014	0.568	0.543	0.594	**< 0.001**
2015	0.411	0.387	0.436	**< 0.001**
2016	0.916	0.856	0.980	**0.0106**
2017	0.773	0.722	0.827	**< 0.001**
2018	1.120	1.003	1.251	**0.0439**
2019	1.133	0.999	1.284	0.0511
**Mild body marks**				
2013	*Intercept*			
2014	0.277	0.268	0.286	**< 0.001**
2015	0.244	0.235	0.254	**< 0.001**
2016	0.210	0.201	0.220	**< 0.001**
2017	0.119	0.113	0.125	**< 0.001**
2018	0.088	0.083	0.094	**< 0.001**
2019	0.049	0.044	0.054	**< 0.001**
**Dirty bodies**				
2013	*Intercept*			
2014	0.741	0.697	0.788	**< 0.001**
2015	0.654	0.605	0.708	**< 0.001**
2016	0.825	0.754	0.904	**< 0.001**
2017	1.037	0.947	1.135	0.435
2018	1.910	1.666	2.191	**< 0.001**
2019	1.816	1.569	2.103	**< 0.001**

Supplementary Table S4 P-values for pair-wise comparison between years with Tukey's HSD for the proportion of pigs requiring hospitalization, lame pigs, pigs with severe tail lesions, pigs with severe body marks and enrichment use ratio.

Pair-wise comparisons between Year1-year2	Pigs requiring hospitalization[1]	Lame pigs[1]	Severe tail lesions[2]	Severe body marks[1]	Enrichment use ratio[2]
2014 - 2013	**< 0.001**	**<0.001**	**< 0.001**	1.0000	0.152
2015 - 2013	**< 0.001**	**<0.001**	**0.00243**	**<0.001**	**<0.001**
2016 - 2013	**< 0.001**	**<0.001**	0.30154	**<0.001**	**<0.001**
2017 - 2013	**< 0.001**	**<0.001**	0.99907	**<0.001**	0.370
2018 - 2013	**< 0.001**	**<0.001**	**0.00711**	**<0.001**	**<0.001**
2019 - 2013	**< 0.001**	**<0.001**	**< 0.001**	**<0.001**	**<0.001**
2015 - 2014	**< 0.001**	**<0.001**	1.00000	**<0.001**	**<0.001**
2016 - 2014	**< 0.001**	**<0.001**	1.00000	**<0.001**	**<0.001**
2017 - 2014	**< 0.001**	**<0.001**	1.00000	**<0.001**	0.968
2018 - 2014	**< 0.001**	**<0.001**	1.00000	**<0.001**	**<0.001**
2019 - 2014	**< 0.001**	**<0.001**	0.46530	**<0.001**	**<0.001**
2016 - 2015	**< 0.001**	**<0.001**	1.00000	**<0.001**	0.998
2017 - 2015	**< 0.001**	**<0.001**	1.00000	**<0.001**	1.000
2018 - 2015	**< 0.001**	1.0000	0.99996	**<0.001**	**<0.001**
2019 - 2015	**< 0.001**	**<0.001**	**< 0.001**	**<0.001**	**<0.001**
2017 - 2016	0.99997	0.8095	1.00000	**<0.001**	1.000
2018 - 2016	0.37498	1.0000	0.22093	**<0.001**	**<0.001**
2019 - 2016	**0.00299**	**0.0303**	**< 0.001**	**<0.001**	**<0.001**
2018 - 2017	0.22093	1.0000	**< 0.001**	0.5405	**<0.001**
2019 - 2017	**< 0.001**	0.2393	**< 0.001**	**0.0136**	**<0.001**
2019 - 2018	0.24065	**<0.001**	**< 0.001**	0.4344	1.000

[1] *pair-wise comparison: if P<0.05 year1<year2*

[2] *pair-wise comparison: if P<0.05 year1>year2*

Supplementary Table S5 P-values for pair-wise comparison between years with Tukey's HSD for the proportion of pigs with mild tail lesions, dirty tails, mild body marks, dirty bodies.

Pair-wise comparisons between year1-year2	Mild tail lesions [1]	Dirty tails [2]	Mild body marks [1]	Dirty bodies [2]
2014 - 2013	< **0.001**	1.00000	< **0.001**	1.0000
2015 - 2013	< **0.001**	1.00000	< **0.001**	1.0000
2016 - 2013	< **0.001**	1.00000	< **0.001**	1.0000
2017 - 2013	**0.00142**	1.00000	< **0.001**	0.8751
2018 - 2013	< **0.001**	0.22053	< **0.001**	**<0.001**
2019 - 2013	< **0.001**	0.24868	< **0.001**	**<0.001**
2015 - 2014	**0.03356**	1.00000	< **0.001**	1.0000
2016 - 2014	< **0.001**	< **0.001**	< **0.001**	0.0766
2017 - 2014	1.00000	< **0.001**	< **0.001**	**<0.001**
2018 - 2014	< **0.001**	< **0.001**	< **0.001**	**<0.001**
2019 - 2014	< **0.001**	< **0.001**	< **0.001**	**<0.001**
2016 - 2015	**0.00458**	< **0.001**	< **0.001**	**<0.001**
2017 - 2015	1.00000	< **0.001**	< **0.001**	**<0.001**
2018 - 2015	< **0.001**	< **0.001**	< **0.001**	**<0.001**
2019 - 2015	**0.00140**	< **0.001**	< **0.001**	**<0.001**
2017 - 2016	1.00000	1.00000	< **0.001**	**<0.001**
2018 - 2016	0.13620	**0.00484**	< **0.001**	**<0.001**
2019 - 2016	0.42814	**0.01062**	< **0.001**	**<0.001**
2018 - 2017	< **0.001**	< **0.001**	< **0.001**	**<0.001**
2019 - 2017	< **0.001**	< **0.001**	< **0.001**	**<0.001**
2019 - 2018	0.99995	0.98993	< **0.001**	1.0000

[1] Pair-wise comparison: if P<0.05 year1<year2

[2] Pair-wise comparison: if P<0.05 year1>year2

Supplementary Table S6 Changes over seasons compared to winter for the mandatory welfare outcomes and enrichment use ratio: Odds ratio, confidence intervals and P-value for all pens included in the study.

	Odd ratio	CI 95%		P-value
**Pigs requiring hospitalization**				
Winter	*Base level*			
Autumn	1.105	1.018	1.198	**0.016**
Spring	0.873	0.801	0.952	**0.002**
Summer	0.944	0.869	1.026	0.177
**Lameness**				
Winter	*Base level*			
Autumn	0.961	0.917	1.008	0.101
Spring	1.255	1.198	1.314	< **0.001**
Summer	0.856	0.816	0.899	< **0.001**
**Severe tail lesions**				
Winter	*Base level*			
Autumn	1.224	1.155	1.297	< **0.001**
Spring	1.166	1.098	1.239	< **0.001**
Summer	1.033	0.973	1.096	0.293
**Severe body marks**				
Winter	*Base level*			
Autumn	0.946	0.897	0.999	**0.044**
Spring	1.042	0.987	1.101	0.138
Summer	0.987	0.936	1.042	0.640
**Enrichment use ratio**				
Winter	*Base level*			
Autumn	0.850	0.780	0.925	< **0.001**
Spring	0.668	0.612	0.730	< **0.001**
Summer	0.633	0.582	0.690	< **0.001**

Supplementary Table S7 values for pair-wise comparison between seasons with Tukey's HSD for the proportion of pigs requiring hospitalization, lame pigs, pigs with severe tail lesions, pigs with severe body marks and enrichment use ratio.

Pair-wise comparisons between seasons	Pigs requiring hospitalization[2]	Lame pigs[2]	Severe tail lesions[1]	Severe body marks[1]	Enrichment use ratio[2]
autumn - spring	**< 0.001**	1.000	1.0000	**<0.001**	**<0.001**
summer - spring	0.15703	1.000	**<0.001**	0.101	1.0000
winter - spring	**0.00565**	1.000	**<0.001**	0.276	**<0.001**
summer - autumn	1.00000	1.000	**<0.001**	1.000	1.0000
winter - autumn	1.00000	0.216	**<0.001**	1.000	**<0.001**
winter - summer	0.3365	**<0.001**	0.4907	0.997	**<0.001**

[1] Pair-wise comparison: if P<0.05 year1<year2

[2] Pair-wise comparison: if P<0.05 year1>year2

Supplementary Table S8 Kendall's tau-b correlation coefficient between the average percentages of welfare outcomes (H: pigs requiring hospitalization, L: lame pigs, STL: pigs with severe tail lesions, SBM; pigs with severe body marks, R: enrichment use ratio) for individual farms in each visit (1st to 8th visits). (P<0.05, Values >0.3 represent a significant correlation).

	H1	H2	H3	H4	H5	H6	H7	H8
H1	1.000	0.251	0.175	0.174	0.185	0.139	0.088	0.097
H2		1.000	0.218	0.178	0.141	0.139	0.130	0.123
H3			1.000	0.217	0.170	0.153	0.113	0.105
H4				1.000	0.216	0.155	0.096	0.097
H5					1.000	0.182	0.142	0.113
H6						1.000	0.142	0.099
H7							1.000	0.203
H8								1.000
	L1	L2	L3	L4	L5	L6	L7	L8
L1	1.000	0.293	0.254	0.255	0.266	0.189	0.148	0.138
L2		1.000	**0.321**	**0.321**	0.293	0.269	0.230	0.180
L3			1.000	**0.366**	**0.308**	**0.285**	0.246	0.214
L4				1.000	**0.362**	**0.331**	**0.303**	0.223
L5					1.000	**0.376**	**0.304**	0.254
L6						1.000	**0.389**	**0.331**
L7							1.000	**0.368**
L8								1.000
	STL1	STL2	STL3	STL4	STL5	STL6	STL7	STL8
STL1	1.000	0.208	0.192	0.157	0.192	0.199	0.137	0.116
STL2		1.000	0.206	0.174	0.185	0.166	0.144	0.104
STL3			1.000	0.246	0.219	0.195	0.155	0.181
STL4				1.000	0.248	0.188	0.172	0.159
STL5					1.000	0.249	0.174	0.122
STL6						1.000	0.264	0.202
STL7							1.000	0.228
STL8								1.000
	SBM1	SBM2	SBM3	SBM4	SBM5	SBM6	SBM7	SBM8
SBM1	1.000	0.279	0.259	0.275	0.190	0.193	0.184	0.194
SBM2		1.000	0.279	0.234	0.186	0.184	0.158	0.148
SBM3			1.000	**0.358**	**0.307**	0.296	0.273	0.229
SBM4				1.000	**0.378**	**0.315**	**0.303**	0.270
SBM5					1.000	**0.288**	**0.292**	**0.301**
SBM6						1.000	**0.332**	**0.352**
SBM7							1.000	**0.358**
SBM8								1.000
	R1	R2	R3	R4	R5	R6	R7	R8
R1	1.000	**0.304**	**0.425**	0.180	0.029	0.123	-0.077	**0.564**
R2		1.000	**0.557**	**0.762**	**0.441**	0.042	0.121	0.294
R3			1.000	**0.602**	**0.276**	-0.017	**0.382**	0.286
R4				1.000	**0.441**	0.074	0.154	**0.667**
R5					1.000	**0.343**	**0.363**	**0.378**
R6						1.000	**0.219**	**0.333**
R7							1.000	0.091

Supplementary Table S9 Annual mean and calculated margin of error considering the sampling size of each year (accounting for the design effect) and 80% power for all welfare outcomes.

	Pigs requiring hospitalization	Lame pigs	Severe tail lesions	Severe body marks	Enrichment use ratio	Mild tail lesions	Mild body marks	Dirty tails	Dirty bodies
Mean 2013	0.14	0.34	0.16	0.29	47.1	1.76	13.09	5.77	3.06
Mean 2014	0.10	0.22	0.20	0.34	49.8	0.98	5.36	3.27	2.17
Mean 2015	0.06	0.16	0.16	0.25	43.9	0.68	5.35	3.99	2.76
Mean 2016	0.04	0.11	0.15	0.20	50.3	0.61	5.74	5.85	3.61
Mean 2017	0.03	0.10	0.14	0.13	39.4	0.87	5.19	8.31	4.59
Mean 2018	0.03	0.14	0.15	0.12	64.1	0.42	3.74	4.68	4.68
Mean 2019	0.03	0.09	0.20	0.11	64.1	0.56	1.74	4.77	4.82
Margin of error 2013	0.041	0.081	0.080	0.113	2.31	0.26	0.71	0.92	0.75
Margin of error 2014	0.027	0.038	0.072	0.085	6.50	0.32	0.80	1.23	1.04
Margin of error 2015	0.021	0.033	0.055	0.071	8.55	0.30	1.06	1.69	1.51
Margin of error 2016	0.029	0.031	0.063	0.072	9.88	0.45	1.15	2.62	1.96
Margin of error 2017	0.028	0.028	0.055	0.052	17.65	0.82	1.25	3.72	2.53
Margin of error 2018	0.017	0.048	0.068	0.055	30.97	0.40	1.13	3.32	3.36
Margin of error 2019	0.022	0.024	0.075	0.044	4.42	0.72	0.99	3.97	4.02
